# Effectiveness of a clinical decision support system with prediction modeling to identify patients with health-related social needs in the emergency department: Study protocol

**DOI:** 10.1371/journal.pone.0323094

**Published:** 2025-05-12

**Authors:** Olena Mazurenko, Christopher A. Harle, Justin Blackburn, Nir Menachemi, Adam Hirsh, Shaun Grannis, Malaz Boustani, Paul I. Musey, Titus K. Schleyer, Lindsey M. Sanner, Joshua R. Vest

**Affiliations:** 1 Department of Health Policy & Management, Richard M. Fairbanks School of Public Health, Indiana University Indianapolis, Indianapolis, Indiana, United States of America; 2 Regenstrief Institute, Indianapolis, Indiana, United States of America; 3 Department of Psychology, School of Science, Indiana University Indianapolis, Indianapolis, Indiana, United States of America; 4 Center for Health Innovation and Implementation Science, School of Medicine, Indiana University, Indianapolis, Indiana, United States of America; 5 Department of Emergency Medicine, School of Medicine, Indiana University, Indianapolis, Indiana, United States of America; PLOS: Public Library of Science, UNITED KINGDOM OF GREAT BRITAIN AND NORTHERN IRELAND

## Abstract

**Introduction:**

Health-related social needs (HRSNs) encompass various non-medical risks from a patient’s life circumstances. The emergency department (ED) is a crucial yet challenging setting for addressing patient HRSNs, a clinical decision support (CDS) intervention could assist in identifying patients at high risk of having HRSNs. This project aims to implement and evaluate a CDS intervention that offers ED clinicians risk prediction scores to determine which patients will likely screen positive for one or more HRSNs.

**Materials & methods:**

The FHIR-based CDS intervention, implemented in the ED setting of a health system in Indianapolis, Indiana, will use health information exchange data to generate logit-derived probability scores that estimate an adult patient’s likelihood of screening positive for each of the following HRSNs: housing instability, food insecurity, transportation barriers, financial strain, and history of legal involvement. For each HRSN, ED clinicians will have access to the patient’s likelihood of screening positive categorized as “high,” “medium,” or “low” based on tertiles in the distribution of each likelihood score. Clinician participation in the CDS will be voluntary. The intervention’s effects will be assessed using a difference-in-difference approach with a pre-post design and a propensity-matched comparison group of ED patients from the same metropolitan area. Outcomes of interest include whether a formal HRSN screening was conducted, whether a referral was made to an HRSN service provider (e.g., social worker), and whether a repeat ED revisit (at 3, 7, and 30 days) or primary care follow-up (within 7 days) occurred.

**Discussion:**

Efficiently and accurately identifying patients with HRSNs could help link them to needed services, improving outcomes and reducing healthcare costs. This protocol will contribute to a growing body of research on the role of CDS interventions in facilitating improved screenings and referrals for HRSNs.

**Trial registration:**

Clincialtrials.gov NCT06655974

## Introduction

Health-related social needs (HRSNs) include various risks from a patient’s life circumstances [1]. Often referred to as social determinants of health, HRSNs are individual-level characteristics such as financial strain, housing instability, and food insecurity [2]. Growing evidence suggests that HRSNs contribute to higher morbidity [3], mortality [4], healthcare utilization [5], disparities [6], and costs [7] in the United States (US) healthcare system. Screening and addressing patients’ HRSNs are becoming an integral part of care delivery. The Centers for Medicare & Medicaid Services mandated HRSN screening via quality reporting metrics for Medicare beneficiaries admitted for inpatient care under the Hospital Inpatient Quality Reporting program [8] and in the outpatient setting through the Merit-based Incentive Payment System [9]. Furthermore, clinicians can be reimbursed annually for HRSNs assessments as part of evaluation & management visits [10]. Current US policy views screening for HRSNs as a means to improve health equity [11], support value-based care [12], and align the healthcare and social care systems [13]. In addition, HRSN screening is a quality measure in the Healthcare Effectiveness Data and Information Set [14] and is consistent with accreditation requirements of the Joint Commission [15]. Finally, HRSN screening is supported by numerous professional associations [16].

Despite broad support for HRSN screening by the above stakeholder groups, most focus has been on inpatient and primary care settings. The emergency department (ED) is also a critical setting for HRSN screening for multiple reasons. First, the majority of ED patients report one or more HRSNs [17,18]. Second, ED clinicians view addressing HRSNs as an opportunity to improve care delivery [19,20] because HRSNs can inhibit treatment adherence and impede follow-up with primary and specialty care [21,22]. Third and last, the ED is an important source of primary and preventative care for many under-resourced and underserved patient populations [23,24]. Thus, efficiently identifying at-risk patients enables to intervene and address patients’ HRSNs [25]. Problematically, several barriers contribute to the lack of HRSNs screening in EDs [20,26] including clinician time constraints, [27] unclear workflows, [20] biases that influence which patients are, or are not, receive formal HRSN screening, [17] and patients’ reluctance to answer certain screening questions [28,29]. While some patients feel comfortable discussing HRSNs with clinicians [30,31], others report that questions feel “too personal” [32] and thus decline to answer questions perceived as stigmatizing and/or unrelated to their clinical situation [32,33]. As a result, a recent Society for Academic Emergency Medicine consensus panel called for more ED-focused research on identifying better approaches to HRSN screening and usage [34,35].

The combined strengths of risk prediction scoring and computerized clinical decision support (CDS) are well-suited to address HRSN screening challenges in the ED. Risk prediction scoring can leverage the growing longitudinal data from electronic health records (EHRs) and health information exchange (HIE). This data provides a comprehensive view of the patient and is not reliant on a single organization for collection. CDS is an ideal delivery mechanism for automatically generated risk prediction scores; it alerts clinicians electronically about relevant patient information [36] and enhances clinical and care processes [37]. Through standards-based communication protocols like FHIR (Fast Healthcare Interoperability Resources), CDS using HIE data can be displayed within an EHR, facilitating clinician access to valuable patient information. A HRSN-focused CDS that automates the risk scoring process could alleviate time constraints, workflow issues, implicit biases, patient reluctance to participate, and other challenges that hinder the implementation of HRSN screening in the ED. Automated risk screening within the CDS could encourage clinicians to offer formal screening tools and refer patients to services addressing their HRSNs.

### Objectives & Hypotheses

This project will implement and evaluate a CDS intervention that informs ED clinicians about which patients are likely to screen positive for an HRSN. Identifying patients with HRSNs is the essential first step in effectively addressing HRSN [38,39]. Currently, in the ED, patients with HRSNs are often under-identified. Identifying these patients enhances clinician awareness, allowing them to provide patient-centered care by connecting patients to the necessary services and resources to address HRSN [6]. Ultimately, key objectives for healthcare organizations in identifying patients with HRSNs are to reduce preventable utilization and increase primary care utilization. Providing services that address HRSNs has effectively reduced repeat utilization among high-risk ED patients [40].

The overall objective of this study is to improve patient care by improving the management of HRSNs. This study will test for effectiveness of the CDS intervention at multiple points in the process from seeking care to changes in utilization with the following hypotheses:

Hypothesis 1: Implementing a CDS intervention will increase the percentage of ED patients screened for HRSNs.

Hypothesis 2: Implementing a CDS intervention will increase the percentage of ED patients referred to HRSN services (based on risk prediction scores).

Hypothesis 3: Implementing a CDS intervention will reduce the percentage of ED patients with ED revisits (measured at 3, 7, and 30 days).

Hypothesis 4: Implementing a CDS intervention will increase the percentage of ED patients who have follow-up visits with primary care providers within 7 days of discharge.

## Materials & methods

### Study design

**Fig 1:**
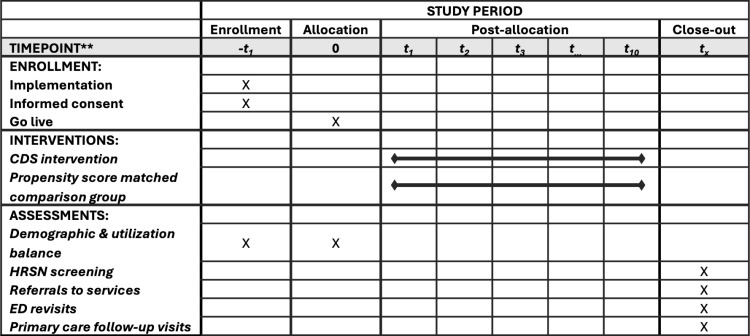
SPIRIT schedule of enrollment, interventions, and assessments.

Study hypotheses will be tested in a non-randomized pre-post design with a comparison group of propensity-score matched ED patients from the same metropolitan area who were unexposed to the CDS intervention. The analytic strategy will follow a difference-in-difference (DiD) approach. This protocol has been developed following the Standard Protocol Items: Recommendations for Interventional Trials (SPIRIT) checklist [41] (See S1 Checklist & S1 File). The SPIRIT schedule and overview are presented as Fig 1.

### Setting

The Indianapolis metropolitan statistical area (MSA) includes eleven counties in central Indiana. With a population of 2.1 million, it is the 34^th^ largest MSA in the US. The MSA population is predominantly White non-Hispanic (67%). Black non-Hispanic (15%) and Hispanic (9%) are the next largest race and ethnicity groups. Median household, educational attainment, and poverty rates are similar with or slightly better than national averages [42].

The intervention site will be Indiana University (IU) Health’s Methodist Hospital ED in downtown Indianapolis. IU Health Methodist is a 1,300-bed non-profit community hospital with a range of tertiary services including: Level 1 trauma Center, orthopedics, cardiovascular, neurosciences, and outpatient surgery. IU Methodist has 100,000 ED visits annually. The Indiana University School of Medicine Department of Emergency Medicine faculty staff the ED. Sixty-one physicians, five fellows, 24 advanced practice providers, and 73 ED residents deliver care at IU Health Methodist (Total = 163). They are supported by 145 nurses and seven social workers and community health workers. Only clinicians practicing at the ED will have access to the CDS intervention.

Comparison patients will be drawn from other IU Health Hospitals within the Indianapolis MSA (n = 3) and Eskenazi Health Hospital. Located near IU Health Methodist, Eskenazi Health is one of the nation’s five largest safety-net health systems and includes a 336-bed hospital with an ED. The comparison group will be chosen from these hospitals to ensure geographic similarity with the intervention group and because we can access screening and primary care data from their EHRs. Neither the intervention nor the potential ED sites had introduced systematic screening for HRSNs. Patients for the comparison group will be drawn during the same pre- and post-test periods as patients from the intervention group.

### Eligibility

#### Clinicians (end-users).

The inclusion criteria are full-time and part-time ED clinicians (physicians, fellows, advanced practice providers, and residents) at IU Health Methodist with access to Health Dart. The exclusion criteria are non-clinicians and those without access to Health Dart.

#### Patients.

The study will focus on adults (≥18 years old) seeking ED care at IU Health Methodist or comparable EDs in Indianapolis, IN MSA. We restrict the study to adults to align with the HRSNs activities and procedures already established at IU Health. Furthermore, our risk score prediction model estimates a positive HRSN score was designed for adults. Based on advice from our clinical partners, we will exclude encounters involving patients with critical illnesses or injuries (e.g., severe trauma patients or those classified as Emergency Severity Index (ESI) level 1) from the analysis. HRSNs hold less clinical priority in these cases, and our CDS intervention is unlikely to be utilized. We will also exclude patients who have been transferred from another inpatient facility or died during the ED encounter. Additionally, since HRSN screening is a required quality metric for inpatients [43], the HRSNs of patients likely to be admitted may be less significant during ED visits. Furthermore, hospital admissions may lead to additional discharge planning activities that could influence subsequent utilization outcomes. Therefore, we will exclude patients ultimately admitted to the hospital during their ED visits from our analysis. Notably, the categories of excluded patients represent a relatively small portion of all ED visits, as most ED visits result in discharges to home [23].

### Ethical considerations

The trial received approval from the Indiana University IRB (#2011558232) and was registered with clinicaltrials.gov (NCT06655974) (See S1 Appendix). The study uses secondary data already collected as part of care and used in research. The CDS intervention has several key features: it is designed for clinicians; end-users can access the Health Dart platform outside of this study context; it functions at a facility level; and it does not disrupt end users’ workflows with prompts or alerts. End users (i.e., clinicians at the intervention site ED) must consent to participate in the study. The clinician subjects will receive the Study Information Sheet in person during a meeting for ED faculty and staff or via email if they are absent. This sheet includes details about the study, clarifying that participation is voluntary, how long the intervention is expected to last, what will occur, how information will be protected, and who to contact with questions. Clinicians will have the opportunity to ask questions of the study team during the ED meetings and via email throughout the study period.

The primary risks for this study are: 1) a loss of patient confidentiality, 2) potential distress from unnecessary discussions of HRSNs, and 3) additional HRSN screening due to a false positive prediction score from the CDS. Steps to prevent a loss of confidentiality will include using synthetic identifiers instead of patient identifiers and storing all data in Indiana University’s HIPAA-compliant computing environment. Potential distress is not more significant than what is experienced during routine care. The vast majority of patients favor health systems collecting HRSN information [44–46]. The risks associated with a false positive are minimal, as most patients will have at least one HRSN [47]. Furthermore, while screening and services are increasingly becoming standard parts of health care encounters, these activities are voluntary and do not involve medical interventions. This protocol will adhere to Indiana University’s policy on reportable events. Any changes to the protocol will be submitted for approval through the Indiana Institutional Review Board (IRB).

### CDS Intervention

The clinician-facing CDS intervention draws extensively on prior work describing clinician, staff, and patient use of HRSN information in the ED [48], those same stakeholders’ views of prediction modeling of HRSN risk [49], identification and engineering of features relevant to measuring HRSNs [47,50–53], and independent development of the CDS platform [54,55]. At the time of this study protocol submission, IU Health ED sites screened neither universally nor systematically for HRSNs. However, the ED was staffed with social workers to support patients with identified HRSNs.

The CDS intervention will be available to clinical end-users at the IU Health Methodist ED via an electronic platform called Health Dart [54,55]. Health Dart is a FHIR-based CDS that directly integrates information from the Indiana HIE into IU Health’s Cerner EHR. Health Dart is organized as a chief complaint-focused dashboard. Health Dart summarizes recent patient information for several conditions/complaints, such as chest pain, abdominal pain, pregnancy, arrhythmia, and dyspnea. End users select the conditions/complaints to view from the navigational menu. Health Dart is single sign-on and context aware (end users do not have to log in separately from the EHR; the information displayed is for the patient being viewed in the EHR). Health Dart usage is limited to clinical staff and is voluntary.

**Fig 2:**
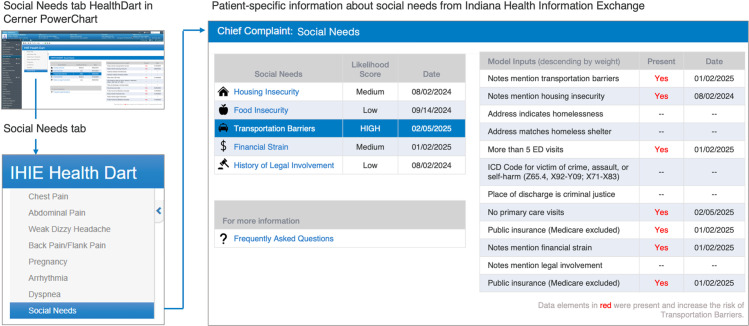
CDS intervention that informs emergency department clinicians which patients are likely to screen positive for a health-related social need.

Our intervention adds the HRSN risk prediction score as an additional condition/complaint displayed in Health Dart (Fig 2).

End users selecting the social needs menu will be presented with a likelihood score for the following HRSNs: housing instability, food insecurity, transportation barriers, financial strain, and history of legal involvement. For each HRSN, we used Lasso models to identify a parsimonious set of predictors for logistic models. Data for prediction models were from the Indiana Network for Patient Care (INPC) database. We summed the unexponentiated post-selection model coefficients to arrive at a total score. Risk prediction scores do not include race and ethnicity as inputs to avoid bias. Model inputs, weights, and performance in development samples are in S2 Appendix For each HRSN, the likelihood of screening positive is reported as “high”, “medium”, or “low” by categorizing the predicted model score. To ensure demographic parity (i.e., the same rate of identifying true positives within each demographic group [56]), we followed prior studies [57–60] by adjusting score thresholds so that the sensitivities (i.e., the percentage of true positives identified by the risk score) were more equivalent within each risk score level and overall.

HRSN risk scores are triggered by patient registration at the ED. Within 20 seconds of patient registration, Indiana HIE passes an HL7 message with patient identifiers to the Regenstrief Institute’s application servers. Backend applications parse the message, apply inclusion criteria, and query the INPC database for model inputs. Model scores are expressed as FHIR resources, which Indiana HIE can use to query when an end user accesses Health Dart. Within Health Dart, after reviewing the overall score, users may select a specific HRSN to open a second panel listing all the features used to derive the score with dates. The HRSN portion of the CDS will only be accessible to clinical staff practicing at the IU Health Methodist ED (the intervention site). Indiana HIE can restrict access based on the roster of User IDs supplied by IU Health. However, clinicians practicing at other IU hospitals and IU Health Methodist could access Health Dart and the HRSN CDS.

### Intervention implementation

The CDS intervention will launch in mid-Spring 2025 and last for nine months. Participant recruitment started on 24/02/2025 and will continue until the end of Spring 2025.

Since the CDS intervention relies on an existing EHR-integrated technology (Health Dart), the implementation will prioritize enhancing end-user education and support regarding the new HRSN component through written materials and in-person demonstrations. We will email written training materials to all ED clinicians (n = 163) at IU Health Methodist two months before the launch, using their work or faculty email addresses. The Department of Emergency Medicine will provide rosters of current clinicians and their contact information to the study team. The written materials will include a slide deck summarizing the study goals, trial dates, IRB approval information, and the IRB study information sheet. The slide deck will also serve as reference material with step-by-step instructions for accessing the HRSN scores with screenshots, guidance on interpreting HRSN scores, and contact information for the study team for assistance.

During the ED faculty and staff meetings, team members will demonstrate how to access Health Dart within the EHR and the HRSN CDS intervention. The demonstrations will use two patient vignettes that draw upon the concepts of Agile Storytelling [61] to create demand for the HRSN CDS among potential end-users. The patient vignettes portray ED clinicians as heroes striving to overcome the challenges of providing adequate care in the presence of HRSN (the “villain”), to patients in need (the “drama”), and how the HRSN CDS could support their efforts (the “resolution”). The vignettes will also demonstrate how to access Health Dart and the HRSN component within the EHR. Three team members (OM, LS, JRV) developed vignettes based on challenging cases shared by ED clinicians in our qualitative interviews [48,49]. A clinician team member checked the vignettes for clinical relevance and accuracy. We also pilot-tested the vignettes with two physician leaders, focusing on transportation barriers and food insecurity (See S3 Appendix with vignettes).

The remainder of the study team presentation will be dedicated to answering end-user questions accompanied by a Frequently Asked Questions (FAQ) document, which will be distributed to all in attendance. The FAQ document already has been integrated into the CDS interface. All training materials will be emailed to end users as part of the meeting’s regular minutes and follow-up communications from department leadership. For faculty absent from meetings or upon request, the team will offer one-on-one training in person or over Zoom as appropriate. Follow-up reminder emails containing condensed training materials will be sent monthly, and research team members will provide study updates and answer questions at quarterly faculty meetings. All activities will be conducted with the support of ED clinical leadership.

### Allocation

The study protocol does not include blinding, masking, or random assignment. While Health Dart is available to every IU Health ED, the CDS intervention using HRSN risk scores will only be active at the IU Health Methodist ED during the study period. The Health Dart platform is accessible to clinical staff for all patient encounters. The CDS intervention will generate scores for all eligible adult patients. If risk scores cannot be produced (due to system outages or the absence of prior patient data), error messages explaining the reasons will be provided to end users. These encounters will be excluded from the analyses.

### Data

The primary data source to generate a patient’s HRSN risk score will be the INPC database, which is a statewide HIE. INPC database includes over 67 million diagnostic studies, procedure results, operative notes, discharge summaries, and radiology images [62]. The INPC comprises a statewide master patient index, enabling medical record linkage across institutions. The INPC also includes clinical notes, to which we will apply our previously published natural language processing (NLP) algorithms to identify records of each of the aforementioned HRSNs from all available note types [63,64]. Additionally, we will link patients’ most recent address to the Area Deprivation Index Scores, which is a composite measure that describes small area socioeconomic status [65]. We will also match addresses to lists of known homeless shelters, aliases for homelessness, and locations associated with the justice system.

The INPC data will be supplemented by extracts from included health systems’ (e.g., IU Health and Eskenazi Health) EHR systems. These additional sources will provide information on study outcome measures about HRSN screening results, which are not shared as part of the INPC. IU Health uses the PRAPARE screening tool [66], which is recorded in the EHR. Eskenazi Health uses the screening tool included in the Epic EHR, which is also recorded and accessible to our study team.

All end-user interactions with Health Dart and CDS intervention activities will be stored in system log files. The system log files will provide information on modeling results and CDS intervention usage.

### Data management

#### Research data.

The project will result in patient clinical data measured at the encounter-level and derived from a combination of information extracted from EHR, INPC, and HIE user log files. All sources are secondary data sources. All data will be stored and analyzed within the Indiana University High Performance Computing Environment. The environment meets requirements established in the HIPAA Security Rule thereby enabling its use for work involving data that contain protected health information. Indiana University official policy sets forth stringent standards for managing access, maintaining data integrity and security, manipulating and extracting data for reports, and choosing appropriate locations and methods for storage of health data and protected health information.

#### Metadata, other relevant data, and associated documentation.

For all data sources, the research team will record the following metadata elements using the standard template (in a spreadsheet). The template will include the data sources (INPC, EHR, or HIE), extraction dates, formats (e.g., CSV), source table names, descriptions, and the names of individuals extracting the data for tracking purposes.

#### Related tools, analytic software and/or code.

All analytic software code maybe a combination of SAS software, R scripts, or Stata programs. These tools will be used to manipulate, clean, and analyze the data.

#### Data and associated research product preservation.

All research data, analytic code, and metadata produced during the project will be preserved for at least three-years from the data of the first study publication or the end of the trial, whichever is later. At the end of the study period, all datasets will be removed of personal identifiers and reviewed by our honest data broker. Long-term storage of all research files and associated data will be within the Indiana University Scholarly Data Archive (SDA; https://pti.iu.edu/storage/sda), a distributed storage service that is centrally supported across mirrored tape silos in Bloomington and Indianapolis. Storage will be managed by the study PI.

### Primary outcomes

1. Percent of ED encounters screened for HRSNs. The numerator will be an ED encounter with any indication of HRSN screening using any tool or questionnaire, regardless of patient completion or results. The denominator will be all eligible ED encounters (see **Inclusion criteria**, above). IU Health uses the PRAPARE screening tool [66], which is recorded in the EHR. Eskenazi Health uses the screening tool included in the Epic EHR, which is also recorded and accessible to our study team.
2. Percent of ED encounters that were referred for HRSN services. The numerator will be ED encounters with a referral to social worker, case management, community health workers, or related services within 24 hours of the ED encounter (see S4 Appendix). The denominator will be all eligible ED encounters (see **Inclusion criteria**, above).

### Secondary outcomes

3a Percent of encounters with an ED revisit measured at 3 days. The numerator will be an ED encounter at any facility included in the INPC database within 3 days of an ED encounter at an intervention or comparator site. ED revisits may serve as the index visit for subsequent revisits. The denominator will be all eligible ED encounters (see **Inclusion criteria**, above). Encounters resulting in an inpatient admission will be excluded from the numerator and denominator.
3b Percent of encounters with an ED revisit measured at 7 days. The numerator will be an ED encounter at any facility included in the INPC database within 7 days of an ED encounter at an intervention or comparator site. ED revisits may serve as the index visit for subsequent revisits. The denominator will be all eligible ED encounters (see **Inclusion criteria**, above). Encounters resulting in an inpatient admission will be excluded from the numerator and denominator.
3c Percent of encounters with an ED revisit measured at 30 days. The numerator will be an ED encounter at any facility included in the INPC database within 30 days of an ED encounter at an intervention or comparator site. ED revisits may serve as the index visit for subsequent revisits. The denominator will be all eligible ED encounters (see **Inclusion criteria**, above). Encounters resulting in an inpatient admission will be excluded from the numerator and denominator.
4 Percent of ED encounters with primary care visit within 7 days of an ED encounter. The numerator will include all ED encounters with a completed family medicine, internal medicine, OBGYN, or geriatrician visit [64] within 7 days of the ED visit. The denominator will be all eligible ED encounters (see **Inclusion criteria**, above).

### Other pre-specified outcome measures

1. Percent of ED encounters where the HRSN CDS intervention was accessed. The numerator will include encounters with access of the social needs section (containing the risk prediction scores) of the Health Dart CDS during the study visit (defined as within 24 hours). Access will be defined as record of the end user visiting the HRSN page in the system user logs. The denominator will be all eligible ED encounters (see **Inclusion criteria**, above). Limited to the intervention site only.
2. Percent of ED encounters where the CDS platform (Health Dart) was accessed. It is possible that our study may increase usage of the overall CDS platform, but not the HRSN intervention portion. The numerator will include encounters with access of CDS platform (Health Dart) during the study visit (defined as within 24 hours). Access will be defined as record of the end user visiting initiating a request to the Health Dart application from the EHR. Any portion of the Health Dart platform (not just the health-related social needs section) is included. The denominator will be all eligible ED encounters (see **Inclusion criteria**, above). Limited to the intervention site only.

### Covariates & Other Variables

Patients will be described by categorized age (18–29; 30–49, 50–64; and over 65), a binary indicator for female sex (compared to all others), a single race and ethnicity categorical variable following Office of Management & Budget guidelines (i.e., Black non-Hispanic, Hispanic, Multirace/Other/Unknown, White non-Hispanic), counts of the total number of inpatient admissions, ED visits, primary care visits, behavioral health visits, and specialist visits in the past 12 months, and Elixhauser comorbidity index scores [67]. We will also extract previous HRSN screening results and ICD-10 Z codes for social needs from the past 12 months. We will also extract (as binary indicators) mentions of HRSNs from clinical notes included in the past 12 months from INPC data. A set of deterministic, rule-based NLP algorithms, nicknamed Finding Other Risks & Contexts Electronically, will identify mentions of financial strain, housing instability, food insecurity, legal problems, and unemployment [63]. These algorithms were developed and evaluated using data from three health systems in Indiana and Florida [64]. Using encounter location, we will classify each screening, ICD-10 Z code, and clinical note as associated with inpatient, ED, or primary care.

From the CDS system logs we will extract the prediction model results for each ED encounter at the intervention site. For the comparators, we will be able to replicate the risk prediction model results using the data described above. From the CDS system logs, we will also be able to extract specific user behaviors: access of the Health Dart CDS and access of the social needs section (containing the risk prediction scores) during the study visit (defined as within 24 hours).

### Propensity score weighting

To address selection bias that may occur based on the non-random assignment of the intervention site, propensity scores will be estimated following the approach described by Stuart and colleagues using multinomial logit models to weight all observations to the intervention group in the pre-intervention period [68]. In this approach, we will use a categorical variable describing each treatment and observation combination (i.e., pre-treatment, post-treatment, pre-comparison, and post-comparison). The pre-treatment will be the baseline category. Because we use a DiD analytic approach, we cannot use any variables in the propensity score matching that will potentially be affected by the intervention [68]. This means that propensity score modeling will exclude the number of ED visits and HRSN screenings related to our outcome measures. This exclusion requirement potentially extends to HRSNs recorded using ICD10 Z codes or in notes as the intervention could prompt additional documentation. Therefore, we will compare these variables at baseline and explore including ICD10 Z codes and HRSNs documented in notes from non-emergency department settings as covariates for adjustment in the final models.

Modeling will draw on the remaining covariates identified above. We will not consider interaction terms. We will use a backwards stepwise elimination approach to arrive at a best fitting model and report overall model fit statistics [69]. We will check models for balance using means and standardized differences. We will iterate on model selection until balance is achieved. From the final model, we will obtain the predicted probability of each observation being in each treatment and observation combination group of the categorical variable. To arrive at a weight reflective of the probability of being in the pre-treatment group, we will divide the predictive probability of being in the pre-treatment group by the probability of being in the observation’s actual group. All pre-treatment group observations will have a weight of 1. Given potential heterogeneity across ED settings, we will explore the potential impact of each ED on the sample. However, as none of the EDs has a systematics HRSN screening program, differences are likely due to patient characteristics which will be matched/weighted by the propensity score.

### Statistical analyses

We will describe the sample using proportions and means. Our primary analysis will be a DiD approach comparing patient-encounter outcomes at the CDS intervention site with a propensity score weighted sample from other EDs. As our primary strategy we will adopt an intention to treat analysis where all eligible encounters at the intervention ED site will be considered as having been exposed to the treatment regardless of actual use of the CDS.

The following weighted regression model will estimate the probability of each outcome:

${Outcome}_{it}=\alpha_{0}+\;\beta_{1}{Treat}_{i}+\;\gamma {Post}_{t}+\;\delta_{rDD}\left({Treat}_{i}*{Post}_{t}\right)+Z_{it}+\varepsilon_{it}$

Patient-encounters are indexed by i and time by t. ${Outcome}_{it}$ is a binary indicator representing each study outcome. ${Treat}_{i}$ is a binary indicator of inclusion in the CDS intervention group, i.e., patient- encounters at IU Health Methodist ED. ${Post}_{t}$ is a dummy variable indicating all post-intervention go-live months. $\delta_{rDD}\;$is the DiD estimator and will test our hypotheses that the CDS intervention is associated with each outcome. $Z_{it}$ is a set of patient-level, time varying adjustment covariates. $\varepsilon_{it}$ is the error term. Standard errors will be robust clustered at the highest level feasible; specifically, we will check for clustering at the clinician and patient levels and adjust accordingly. As described above, all observations will be weighted using the propensity score derived weights. We will compare the weighted model with the unweighted model. Given the length of panel, we will also check for, and correct for, autocorrelation as needed.

### Subgroup analyses

We will stratify all analyses using the results of the prediction modeling as this is intended to affect user behavior. Specifically, we will compare outcomes by the “high”, “medium”, and “low” risk categories. Additionally, end user activities may vary by potential HRSN, thus we will stratify by specific HRSNs or the number of different HRSNs identified. Additionally, because our prediction models use historical data, we will stratify results by the number of prior encounters in the previous 12 months to explore the potential role of data availability. Finally, we will examine for possible differences by race, ethnicity, sex, and age.

### Sample size

This study’s sample size is fixed at the number of ED encounters among eligible adult patients at IU Health Methodist and the matched weighted comparison group. Based on historical data, the intervention site served an average of 2,930 adult patients monthly. According to national estimates, we assumed approximately 15% of these visits would be excluded based on disposition to inpatient admission, a transfer, or death [23] or were at an Emergency Severity Index level 1 [70]. In the past 12 months (01DEC2023–30NOV2024), the average 7-day revisit rate for the intervention site was 5.5% and 30-day revisit rate was 10.5%. These were the only outcomes available for preliminary analyses and served as the range of our outcomes. We used PASS version 24’s test for two proportions in a repeated design [71] to estimate the power for the above parameters to detect an odds ratio of between 0.90 to 0.50. We assumed 18 time points (9 pre and 9 post) and compound symmetry.

With a monthly sample of 2,400 at the intervention site, we would have >80% power to detect an odds ratio of 0.75 if the readmission rate in the comparison group was 5.5%. For a readmission rate of 10.5%, we could detect an odds ratio of 0.80 with >80% power. Even if we excluded half of the intervention site patients, our sample size would still be sufficient to detect an odds ratio of 0.70 with 80% power.

### Alternative strategies

Alternative strategies may be necessary for defining the sample, applying propensity score matching, and conducting analyses. First, the CDS intervention will be voluntary. If daily access of the CDS (i.e., usage) is insufficient for modeling, we will shift from our proposed intention-to-treat analysis for all patients to a per-protocol analysis. In a per-protocol analysis, we could focus on encounters among clinicians who were consistent or more frequent users of Health Dart, based on historical system log data. While this approach could introduce selection bias, making it our secondary strategy, it would offer insights into the CDS intervention’s associations and outcomes. We would use the non-adopters from the intervention site as the comparison group and generate propensity score weights for this alternative group. Second, we may not achieve balance in our propensity scores. If that occurs, our first choice will be explore the potential effect of our chosen comparator hospitals. We may be forced to draw our sample from a smaller set of hospitals that better match our intervention site’s practice. Alternatively, we could forego weighting and use individual patient covariates in the model or apply a strict matching approach. Additionally, we could consider including other health systems from the state in our comparison group, although this might impact our ability to measure all study outcomes. Finally, our prediction models use data from both EHR and HIE systems, but if EHR data will be unavailable due to technical or administrative reasons, we developed an alternative set of inputs and weights for the prediction score based on the INPC only (HIE database). The prediction target and method remained the same, with adjustments to thresholds to ensure consistent sensitivities across risk score levels (See S5 Appendix).

### Monitoring and auditing

System uptime and end user technical support will be coordinated through the Indiana HIE. The study PI and project manager will review Health Dart usage logs weekly to monitor CDS usage. Ongoing quality assurance checks will be completed throughout the study. Follow-up reminder emails containing training materials will be sent monthly. The team will present updates at faculty meetings throughout the study period.

### Dissemination

We will present our findings at scientific conferences and publish in peer-reviewed journals. The peer-reviewed manuscripts will also be submitted to the National Library of Medicine’s PubMed Central and made publicly available within 12 months of publication, as outlined by the National Institutes of Health Public Access Policy. Furthermore, our media teams at Regenstrief Institute and Indiana University School of Public Health will share study updates and results through social media and print media. Finally, we will present our findings to IUH Methodist ED clinicians and other relevant stakeholders.

## Discussion

Given the high needs faced by many patients, the ED is an important yet complicated setting in which to identify and address HRSNs[35]. Identifying patients with HRSNs is the first, and necessary, step toward improving patient outcomes [16]. This paper describes a protocol for introducing and evaluating a CDS intervention that informs ED clinicians which patients are likely to screen positive for an HRSN. With this focus, the protocol contributes to a rapidly growing body of research on using CDS to support social care and address patients’ HRSNs. These HRSN-focused CDS vary in terms of setting and their use of HRSN screening information. For instance, in primary care settings, CDS have summarized recent screening data to facilitate referrals and service [72,73]. Additionally, in a primary care setting, our own team successfully integrated prediction models into an EHR, which subsequently increased referrals to social service providers in a prospective trial [74]. Similar to this protocol, but in the primary care setting, a trial of a CDS using risk prediction scores is underway [75].

This protocol offers several advantages. First, the DiD analytic strategy will facilitate causal inference regarding the effectiveness of our intervention. The pre-intervention measurement period, along with a propensity score weighted comparison group, protects against secular trends as alternative explanations and accounts for the non-random selection of the intervention site in estimating the effect size. Moreover, the longitudinal design allows us to exclude other quality improvement or ED practice changes as alternative explanations (i.e., any other potential alternative explanations would need to coincide with our intervention). Additionally, we leverage a standards-based, existing CDS platform [54,55] for our HRSN CDS intervention. This platform is already in use and familiar to potential end users.

Nevertheless, the protocol faces some limitations and challenges. Most notably, using the existing platform and, by extension, our CDS intervention will be voluntary and, thus, not universally adopted by the targeted end users. All eligible encounters will have predicted risk scores (if the information is available), but end users are not required to access or act upon the information. Despite this, our CDS intervention holds a strong promise to improve the identification of patients with HRSNs in the ED.

## Supporting information

S1 Checklist SPIRIT Checklist. (DOCX)

S1 File Study Protocol. (DOCX)

S1 Appendix Clinical Trials Protocol. (PDF)

S2 Appendix Model Performance. (DOCX)

S3 Appendix Agile Implementation Vignettes. (DOCX)

S4 Appendix Health-Related Social Needs Service Measures. (DOCX)

S5 Appendix Alternative Model Specifications. (DOCX)
