# Supplementary material for: Effectiveness of a clinical decision support system with prediction modeling to identify patients with health-related social needs in the emergency department: Study protocol
Source: PLoS One. 2025 May 12;20(5):e0323094. doi: 10.1371/journal.pone.0323094 (PMC12068607; doi:10.1371/journal.pone.0323094)
Supplement: S1 File — (DOCX) [file pone.0323094.s002.docx]

Study Protocol

**Scientific Title: Effectiveness of a clinical decision support system with prediction modeling to identify patients with health-related social needs in the emergency department: study protocol**

**Public Title: Protocol: Effectiveness of a health-related social needs clinical decision support system**

**Contact for Public Queries:** Olena Mazurenko, MD, PhD

Health Sciences Building, 1050 Wishard Boulevard, RG 6140, Indianapolis, Indiana, United States, 46202, 317-274-3341

[omazuren@iu.edu](mailto:omazuren@iu.edu)

**Contact for Scientific Queries:** Joshua R. Vest, PhD, MPH

Health Sciences Building, 1050 Wishard Boulevard, RG 6155, Indianapolis, Indiana, United States, 46202, 317-278-8410

[joshvest@iu.edu](mailto:joshvest@iu.edu)

**Funding:** Agency for Healthcare Research & Quality (1R01HS028008; PI: Vest).

**Primary sponsor:** Indiana University (responsibilities: trial registration, initiation, and study management)

**Countries of Recruitment:** United States

**Health Condition(s) or Problem(s) Studied:** Health-related social needs (HRSNs)

**Trial Identifier & Registry Name:** ClinicalTrials.gov Identifier: NCT06655974. Predictive Modeling for Social Needs in Emergency Department Settings. Date of the registration: 10/22/2024

The Indiana University Institutional Review Board approved the study: #2011558232 (Date of approval: 12/14/2020)

**Protocol Version:** 02/19/2025

1. **Rationale & Objectives**

Health-related social needs (HRSNs) encompass various non-medical risks from a patient’s life circumstances. Since the emergency department (ED) is a crucial yet challenging setting for recognizing and addressing patient HRSNs, a clinical decision support (CDS) intervention could assist in identifying patients at high risk of having HRSNs. This project will implement and evaluate a CDS intervention that informs ED clinicians about which patients are likely to screen positive for an HRSN. Identifying patients with HRSNs is the essential first step in effectively addressing HRSN. Currently, in the ED, patients with HRSNs are often under-identified. Identifying these patients enhances clinician awareness, allowing them to provide patient-centered care by connecting patients to the necessary services and resources to address HRSN. Ultimately, key objectives for healthcare organizations in identifying patients with HRSNs are to reduce preventable utilization and increase primary care utilization. Providing services that address HRSNs has effectively reduced repeat utilization among high-risk ED patients.

**Specific Aims and Hypotheses:**

The overall objective of this study is to improve patient care by improving the management of HRSNs. Specifically, this study will test for effectiveness of the CDS intervention at multiple points in the process from seeking care to changes in utilization with the following hypotheses:

*Hypothesis 1: Implementing a CDS intervention will increase the percentage of ED patients screened for HRSNs.*

*Hypothesis 2: Implementing a CDS intervention will increase the percentage of ED patients referred to HRSN services (based on risk prediction scores).*

*Hypothesis 3: Implementing a CDS intervention will reduce the percentage of ED patients with ED revisits (measured at 3, 7, and 30 days).*

*Hypothesis 4: Implementing a CDS intervention will increase the percentage of ED patients who have follow-up visits with primary care providers within 7 days of discharge.*

1. **Eligibility Criteria**

**Clinicians:**

- Inclusion criteria:
  - ED clinician (physician, fellow, advanced practice provider, resident) delivering care at IU Health Methodist.
  - Has access to Health Dart
  - Full and part-time
- Exclusion criteria:
  - Non-clinician
  - No access to Health Dart

**Patients:**

- Inclusion criteria:
  - Adults (≥18 years old) who seek ED care at IU Health Methodist or comparator EDs within the Indianapolis, IN MSA.
- Exclusion criteria:
- Present with a critical illness or injury (e.g., severe trauma patients or those with Emergency Severity Index (ESI) classification level 1).
- Have been transferred from another inpatient facility or died during the ED encounter.
- ED visits resulting in hospital admissions.

Recruitment status: Pending (Participants are not yet being recruited or enrolled at any site)

1. **Study Design**

The study will employ a non-randomized pre-post design with a comparison group of propensity-score matched ED patients from the same metropolitan area who were unexposed to the CDS intervention. The analytic strategy will follow a difference-in-difference (DiD) approach. The study protocol does not include blinding, masking, or random assignment. While Health Dart is available to every IU Health ED, the CDS intervention using HRSN risk scores will only be active at the IU Health Methodist ED during the study period. The Health Dart platform is accessible to clinical staff for all patient encounters. The CDS intervention will generate scores for all eligible adult patients. If risk scores cannot be produced (due to system outages or the absence of prior patient data), error messages explaining the reasons will be provided to end users. These encounters will be excluded from the analyses.

1. **Intervention**

The clinician-facing CDS intervention draws extensively on prior work describing clinician, staff, and patient use of HRSN information in the ED, those same stakeholders’ views of prediction modeling of HRSN risk identification and engineering of features relevant to measuring HRSNs, and independent development of the CDS platform. At the time of this study protocol submission, IU Health ED sites screened neither universally nor systematically for HRSNs. However, the ED was staffed with social workers to support patients with identified HRSNs.

The CDS intervention will be available to clinical end-users at the IU Health Methodist ED via an electronic platform called Health Dart. Health Dart is a FHIR-based CDS that directly integrates information from the Indiana HIE into IU Health’s Cerner EHR. Health Dart is organized as a chief complaint-focused dashboard. Health Dart summarizes recent patient information for several conditions/complaints, such as chest pain, abdominal pain, pregnancy, arrhythmia, and dyspnea. End users select the conditions/complaints to view from the navigational menu. Health Dart is single sign-on and context aware (end users do not have to log in separately from the EHR; the information displayed is for the patient being viewed in the EHR). Health Dart usage is limited to clinical staff and is voluntary.

Our intervention adds the HRSN risk prediction score as an additional condition/complaint displayed in Health Dart. End users selecting the social needs menu will be presented with a likelihood score for the following HRSNs: housing instability, food insecurity, transportation barriers, financial strain, and history of legal involvement. For each HRSN, we used Lasso models to identify a parsimonious set of predictors for logistic models. We summed the unexponentiated post-selection model coefficients to arrive at a total score. Risk prediction scores do not include race and ethnicity as inputs to avoid bias. For each HRSN, the likelihood of screening positive is reported as “high”, “medium”, or “low” by categorizing the predicted model score. To ensure demographic parity (i.e., the same rate of identifying true positives within each demographic group), we followed prior studies by adjusting score thresholds so that the sensitivities (i.e., the percentage of true positives identified by the risk score) were more equivalent within each risk score level and overall.

HRSN risk scores are triggered by patient registration at the ED. Within 20 seconds of patient registration, Indiana HIE passes an HL7 message with patient identifiers to the Regenstrief Institute’s application servers. Backend applications parse the message, apply inclusion criteria, and query the INPC database for model inputs. Model scores are expressed as FHIR resources, which Indiana HIE can use to query when an end user accesses Health Dart. Within Health Dart, after reviewing the overall score, users may select a specific HRSN to open a second panel listing all the features used to derive the score with dates. The HRSN portion of the CDS will only be accessible to clinical staff practicing at the IU Health Methodist ED (the intervention site). Indiana HIE can restrict access based on the roster of User IDs supplied by IU Health. However, clinicians practicing at other IU hospitals and IU Health Methodist could access Health Dart and the HRSN CDS.

1. **Measures and Outcomes**

**Primary outcomes**

1. Percent of ED encounters screened for HRSNs. The numerator will be an ED encounter with any indication of HRSN screening using any tool or questionnaire, regardless of patient completion or results. The denominator will be all eligible ED encounters (see **Inclusion criteria**, above). IU Health uses the PRAPARE screening tool^53^, which is recorded in the EHR. Eskenazi Health uses the screening tool included in the Epic EHR, which is also recorded and accessible to our study team.
2. Percent of ED encounters that were referred for HRSN services. The numerator will be ED encounters with a referral to social worker, case management, community health workers, or related services within 24 hours of the ED encounter (see Appendix B). The denominator will be all eligible ED encounters (see **Inclusion criteria**, above).

**Secondary outcomes**

3a. Percent of encounters with an ED revisit measured at 3 days. The numerator will be an ED encounter at any facility included in the INPC database within 3 days of an ED encounter at an intervention or comparator site. ED revisits may serve as the index visit for subsequent revisits. The denominator will be all eligible ED encounters (see **Inclusion criteria**, above). Encounters resulting in an inpatient admission will be excluded from the numerator and denominator.

3b. Percent of encounters with an ED revisit measured at 7 days. The numerator will be an ED encounter at any facility included in the INPC database within 7 days of an ED encounter at an intervention or comparator site. ED revisits may serve as the index visit for subsequent revisits. The denominator will be all eligible ED encounters (see **Inclusion criteria**, above). Encounters resulting in an inpatient admission will be excluded from the numerator and denominator.

3c. Percent of encounters with an ED revisit measured at 30 days. The numerator will be an ED encounter at any facility included in the INPC database within 30 days of an ED encounter at an intervention or comparator site. ED revisits may serve as the index visit for subsequent revisits. The denominator will be all eligible ED encounters (see **Inclusion criteria**, above). Encounters resulting in an inpatient admission will be excluded from the numerator and denominator.

1. Percent of ED encounters with primary care visit within 7 days of an ED encounter. The numerator will include all ED encounters with a completed family medicine, internal medicine, OBGYN, or geriatrician visit^64^ within 7 days of the ED visit. The denominator will be all eligible ED encounters (see **Inclusion criteria**, above).

**Other pre-specified outcome measures**

1. Percent of ED encounters where the HRSN CDS intervention was accessed. The numerator will include encounters with access of the social needs section (containing the risk prediction scores) of the Health Dart CDS during the study visit (defined as within 24 hours). Access will be defined as record of the end user visiting the HRSN page in the system user logs. The denominator will be all eligible ED encounters (see **Inclusion criteria**, above). Limited to the intervention site only.
2. Percent of ED encounters where the CDS platform (Health Dart) was accessed. It is possible that our study may increase usage of the overall CDS platform, but not the HRSN intervention portion. The numerator will include encounters with access of CDS platform (Health Dart) during the study visit (defined as within 24 hours). Access will be defined as record of the end user visiting initiating a request to the Health Dart application from the EHR. Any portion of the Health Dart platform (not just the health-related social needs section) is included. The denominator will be all eligible ED encounters (see **Inclusion criteria**, above). Limited to the intervention site only.
3. **Statistical Considerations**

**Power and Sample Size.** This study's sample size is fixed at the number of ED encounters among eligible adult patients at IU Health Methodist and the matched weighted comparison group. Based on historical data, the intervention site served an average of 2,930 adult patients monthly. According to national estimates, we assumed approximately 15% of these visits would be excluded based on disposition to inpatient admission, a transfer, or death23 or were at an Emergency Severity Index level 170. In the past 12 months (01DEC2023 to 30NOV2024), the average 7-day revisit rate for the intervention site was 5.5% and 30-day revisit rate was 10.5%. These were the only outcomes available for preliminary analyses and served as the range of our outcomes. We used PASS version 24’s test for two proportions in a repeated design71 to estimate the power for the above parameters and detect an odds ratio of 0.90 to 0.50. We assumed 18 time points (9 pre and 9 post) and compound symmetry.

With a monthly sample of 2,400 at the intervention site, we would have >80% power to detect an odds ratio of 0.75 if the readmission rate in the comparison group was 5.5%. For a readmission rate of 10.5%, we could detect an odds ratio of 0.80 with >80% power. Even if we excluded half of the intervention site patients, our sample size would still be sufficient to detect an odds ratio of 0.70 with 80% power.

1. **Analytic Plan**

We will describe the study sample using proportions and means. Our primary analysis will be a DiD approach comparing patient-encounter outcomes at the CDS intervention site with a propensity score weighted sample from other EDs. As our primary strategy we adopt an intention to treat analysis approach where all eligible encounters at the intervention ED site will be considered as having been exposed to the treatment regardless of actual use of the CDS. The following weighted regression model will estimate the probability of each outcome:

$${Outcome}_{it}=\alpha_{0}+ \beta_{1}{Treat}_{i}+ \gamma{Post}_{t}+ \delta_{rDD}\left( {Treat}_{i}*{Post}_{t} \right)+Z_{it}+\varepsilon_{it}$$

Patient-encounters are indexed by $i$ and time by $t$. ${Outcome}_{it}$ is a binary indicator representing each study outcome. ${Treat}_{i}$ is a binary indicator of inclusion in the CDS intervention group, i.e., patient- encounters at IU Health Methodist ED. ${Post}_{t}$ is a dummy variable indicating all post-intervention go-live months. $\delta_{rDD}$is the DiD estimator and will test our hypotheses that the CDS intervention is associated with each outcome. $Z_{it}$ is a set of patient-level, time varying adjustment covariates. $\varepsilon_{it}$ is the error term. Standard errors will be robust clustered at the highest level feasible; specifically, we will check for clustering at the clinician and patient levels and adjust accordingly. As described above, all observations will be weighted using the propensity score derived weights. We will compare the weighted model with the unweighted model. Given the length of panel, we will also check for, and correct for, autocorrelation as needed.

**Subgroup analyses**

We will stratify all analyses using the results of the prediction modeling as this is intended to affect user behavior. Specifically, we will compare outcomes by the “high”, “medium”, and “low” risk categories. Additionally, end user activities may vary by potential HRSN, thus we will stratify by specific HRSNs or the number of different HRSNs identified. Additionally, because our prediction models use historical data, we will stratify results by the number of prior encounters in the previous 12 months to explore the potential role of data availability. Finally, we will examine for possible differences by race, ethnicity, sex, and age.

**Alternative strategies**

Alternative strategies may be necessary for defining the sample, applying propensity score matching, and conducting analyses. First, the CDS intervention will be voluntary. If daily access of the CDS (i.e usage) is insufficient for modeling, we will shift from our proposed intention-to-treat analysis for all patients to a per-protocol analysis. In a per-protocol analysis, we could focus on encounters among clinicians who were consistent or more frequent users of Health Dart, based on historical system log data. While this approach could introduce selection bias, making it our secondary strategy, it would offer insights into the CDS intervention's associations and outcomes. We would use the non-adopters from the intervention site as the comparison group and generate propensity score weights for this alternative group. Second, we may not achieve balance in our propensity scores. If that occurs, our first choice will be explore the potential effect of our chosen comparator hospitals. We may be forced to draw our sample from a smaller set of hospitals that better match our intervention site’s practice. Alternatively, we could forego weighting and use individual patient covariates in the model or apply a strict matching approach. Additionally, we could consider including other health systems from the state in our comparison group, although this might impact our ability to measure all study outcomes.

**8. Monitoring**

System uptime and end user technical support will be coordinated through the Indiana HIE. The study PI and project manager will review Health Dart usage logs weekly to monitor CDS usage. Follow-up reminder emails containing training materials will be sent monthly. The team will present updates at faculty meetings throughout the study period.

1. **IDP Sharing Statement**

All data generated during the project will be preserved; however, raw and derived data at the patient level will not be publicly posted due to our use of secondary data from privately held electronic health record and health information exchange systems. Because of the data use restrictions established by consortium agreements among the health system partners contributing EHR data to the Indiana Network for Patient Care, patient-level data cannot be shared or disseminated beyond this project. Nevertheless, de-identified derived data at the patient level used in this study may be shared with investigators whose formal requests are approved by the data owners. Requests can be sent to askRDS@regenstrief.org. Access to this data requires investigator support and a signed data access agreement between the Regenstrief Institute and the investigator’s institution.
