# Supplementary material for: Effectiveness of a clinical decision support system with prediction modeling to identify patients with health-related social needs in the emergency department: Study protocol
Source: PLoS One. 2025 May 12;20(5):e0323094. doi: 10.1371/journal.pone.0323094 (PMC12068607; doi:10.1371/journal.pone.0323094)
Supplement: S1 Appendix — (PDF) [file pone.0323094.s003.pdf]

ClinicalTrials.gov Protocol Registration and Results System (PRS) Receipt
Release Date: February 19, 2025

ClinicalTrials.gov ID: NCT06655974

Study Identification

Unique Protocol ID: 2011558232

Brief Title: Predictive Modeling for Social Needs in Emergency Department Settings

Official Title: Protocol for Evaluating the Effectiveness of a Clinical Decision Support System With Prediction Modeling to Identify Patients With Health-related Social Needs in the Emergency Department

Secondary IDs:

Study Status

Record Verification: February 2025

Overall Status: Not yet recruiting

Study Start: March 10, 2025 [Anticipated]

Primary Completion: December 1, 2025 [Anticipated]

Study Completion: December 31, 2025 [Anticipated]

Sponsor/Collaborators

Sponsor: Indiana University

Responsible Party: Principal Investigator
Investigator: Joshua R. Vest, PhD [joshvest]
Official Title: Professor
Affiliation: Indiana University

Collaborators:

Oversight

U.S. FDA-regulated Drug: No

U.S. FDA-regulated Device: No

U.S. FDA IND/IDE: No

Human Subjects Review: Board Status: Approved
Approval Number: 2011558232
Board Name: Indiana University
Board Affiliation:
Phone: (317) 274-8289
Email: irb@iu.edu
Address:

Human Research Protection Program (HRPP)
Office for Research Compliance
Indiana University
986 Indiana Avenue, 5th Floor
Indianapolis, Indiana 46202

Data Monitoring: No

FDA Regulated Intervention: No

Study Description

Brief Summary: The overall objective of this study is to support emergency department management of patients' health-related social needs. This study will measure the impact of a decision support system that informs clinicians about which patients are likely to screen positive for a health-related social need. The system uses statistical models to create a health-related social need risk score for each patient. The main questions, the study aims to answer are:

- Does providing emergency department clinicians with risk scores on health-related social needs increase screening and referral activities?
- Does providing emergency department clinicians with risk scores on health-related social needs change patients' use of healthcare services?

The decision support system with health-related social needs risk scores will be introduced for all adult patients at one emergency department. Screening rates, referrals, and subsequent healthcare encounters will be compared with emergency departments that did not have access to the decision support system.

Detailed Description:

Conditions

Conditions: Emergency Service, Hospital
Social Determinants of Health

Keywords: informatics

Study Design

Study Type: Interventional

Primary Purpose: Health Services Research

Study Phase: N/A

Interventional Study Model: Parallel Assignment
The intervention will be at the ED level using a pre-post design with a matched comparison group.

Number of Arms: 2

Masking: None (Open Label)

Allocation: Non-Randomized

Enrollment: 48000 [Anticipated]

Arms and Interventions

Arms	Assigned Interventions
Experimental: Decision support intervention group<br>Adult ED patients seeking care the ED site with the health-related social needs decision support system live.	Health-related social needs decision support system<br>The clinical decision support intervention will present emergency department clinicians at an Indianapolis, IN ED with a likelihood score for an adult patient screening positive for the following health-related social needs (HRSNs): housing instability, food insecurity, transportation barriers, financial strain, and history of legal involvement. For each HRSN, the likelihood of screening positive is reported as "high", "medium", or "low". These categorizations are the product of logistic regression models. The clinical decision support intervention will be delivered through an existing FHIR (Fast Healthcare Interoperability Resources) standards-based clinical decision support platform.
No Intervention: Comparison group<br>Adult ED patients created using statistical matching from ED sites in the same metropolitan area.	

Outcome Measures

Primary Outcome Measure:

1. Percent of emergency department encounters screened for health-related social needs (HRSNs)
   The numerator will be an emergency department encounter with any indication of HRSN screening using any tool or questionnaire, regardless of patient completion or results. The denominator will be all eligible ED encounters.

   [Time Frame: During emergency department encounter]
2. Percent of emergency department encounters that were referred for health-related social needs (HRSNs) services
   The numerator will be emergency department encounters with a referral to social worker, case management, community health workers, or related services within 24 hours of the ED encounter. The denominator will be all eligible ED encounters

   [Time Frame: During emergency department encounter]

Secondary Outcome Measure:

3. Percent of encounters with an emergency department revisit measured at 3 days
   The numerator will be an emergency department encounter at any facility included in the Indiana for Network Care database within 3 days of an ED encounter at an intervention or comparator site. ED revisits may serve as the index visit for subsequent revisits. The denominator will be all eligible ED encounters. Encounters resulting in an inpatient admission will be excluded from the numerator and denominator.

   [Time Frame: within 3 days of emergency department encounter]
4. Percent of encounters with an emergency department revisit measured at 7 days
   The numerator will be an emergency department encounter at any facility included in the Indiana for Network Care database within 7 days of an ED encounter at an intervention or comparator site. ED revisits may serve as the index visit for subsequent revisits. The denominator will be all eligible ED encounters. Encounters resulting in an inpatient admission will be excluded from the numerator and denominator.

   [Time Frame: within 7 days of emergency department encounter]
5. Percent of encounters with an emergency department revisit measured at 30 days
   The numerator will be an emergency department encounter at any facility included in the Indiana for Network Care database within 30 days of an ED encounter at an intervention or comparator site. ED revisits may serve as the index visit for subsequent revisits. The denominator will be all eligible ED encounters. Encounters resulting in an inpatient admission will be excluded from the numerator and denominator

[Time Frame: within 30 days of emergency department encounter]

6. Percent of emergency department encounters with primary care visit within 7 days of an ED encounter
   The numerator will include all emergency department encounters with a completed family medicine, internal medicine, OBGYN, or geriatrician visit64 within 7 days of the ED visit. The denominator will be all eligible ED encounters. Encounters resulting in an inpatient admission will be excluded from the numerator and denominator.

   [Time Frame: within 7 days of emergency department visit]

Other Pre-specified Outcome Measures:

7. Percent of emergency department encounters where the health-related social needs (HRSN) decision support system intervention was accessed.
   The numerator will include encounters with access of the social needs section (containing the risk prediction scores) of the decision support system intervention during the study visit (defined as within 24 hours). Access will be defined as record of the end user visiting the HRSN page in the system user logs. The denominator will be all eligible ED encounters. Limited to the intervention site only.

   [Time Frame: At time of emergency department encounter(or within 24 hours)]
8. Percent of emergency department encounters where the clinical decision support platform was accessed
   The numerator will include encounters with access of clinical decision support platform during the study visit (defined as within 24 hours). Access will be defined as record of the end user visiting initiating a request to the decision support application from the EHR. Any portion of the decision support platform (not just the health-related social needs section) is included. The denominator will be all eligible ED encounters (see Inclusion criteria, above). Limited to the intervention site only

   [Time Frame: At time of emergency department encounter(or within 24 hours)]

Eligibility

Minimum Age: 18 Years

Maximum Age:

Sex: All

Gender Based: No

Accepts Healthy Volunteers: Yes

Criteria: Inclusion Criteria:

- Adults (>18 years old)
- Seeking care at Indianapolis, Indiana area emergency departments (EDs).

Exclusion Criteria:

- Children
- Encounters by patients that present with a critical illness/injury (e.g. severe trauma patients or those with Emergency Severity Index (ESI) classification level 1)
- Encounters by patients who have been transferred from another inpatient facility
- Patients that die during the ED encounter
- Encounters among patients who were ultimately admitted during their ED visits from our analysis

Contacts/Locations

Central Contact Person: Joshua R Vest, PhD, MPH
Telephone: 317 278 8410
Email: joshvest@iu.edu

Central Contact Backup:

Study Officials: Joshua R Vest, PhD,MPH
Study Principal Investigator
Indiana University

Locations: **United States, Indiana**
Indiana University Health
Indianapolis, Indiana, United States, 46202
Contact: Joshua Vest joshvest@iu.edu

IPDSharing

Plan to Share IPD: No
All data produced during the project will be preserved, but raw and derived data (at the patient-level) will not be posted publicly because of our use of secondary data from privately held electronic health record and health information exchange systems. Due to the data use restrictions put in place by consortium agreements among the health system partners that contribute EHR data to the Indiana Network for Patient Care, patient-level data cannot be shared or disseminated outside this project. However, de-identified derived data (at the patient-level) used in this study may be shared with investigators whose formal request is approved by the data owners. Requests can be sent to askRDS@regenstrief.org. Access to these data requires investigator support for use and a signed data access agreement between the Regenstrief Institute and the investigator’s institution. #

References

Citations: Mazurenko O, Hirsh AT, Harle CA, McNamee C, Vest JR. Acceptance of Automated Social Risk Scoring in the Emergency Department: Clinician, Staff, and Patient Perspectives. West J Emerg Med. 2024 Jul;25(4):614-623. doi: 10.5811/westjem.18577. PubMed 39028248

Links:

Available IPD/Information:
