# Supplementary material for: Effectiveness of a clinical decision support system with prediction modeling to identify patients with health-related social needs in the emergency department: Study protocol
Source: PLoS One. 2025 May 12;20(5):e0323094. doi: 10.1371/journal.pone.0323094 (PMC12068607; doi:10.1371/journal.pone.0323094)
Supplement: S2 Appendix — (DOCX) [file pone.0323094.s004.docx]

**Appendix S2. HRSN risk prediction model performance**

Table A1. Predictive models in classifying of adult emergency department patients with screening positive for the health-related social needs (HRSN) of housing instability, food insecurity, transportation barriers, financial strain and history of criminal justice involvement using only data available via health information exchange system, Indianapolis, IN.

| Features | Housing instability | Food insecurity | Transportation barriers | Financial strain | History of legal problems |
| --- | --- | --- | --- | --- | --- |
|  | $\beta$ | $\beta$ | $\beta$ | $\beta$ | $\beta$ |
| Address indicates homelessness | 0.2754314* | 0.3661188* | 0.6680268 * | 0.0638165* | 1.1474827* |
| Address matches known shelter address | 0.1110138* |  | 0.5825544 * | 0.0972929* |  |
| Address matches criminal justice location |  |  |  |  | 1.501319 |
| Age | -0.0007814 |  |  |  | 0.0047298* |
| Arrived via taxi at prior ED/hospitalization |  |  | * |  |  |
| Count of behavioral health encounters |  |  | * |  |  |
| Criminal justice associated payer | 0.4349765* |  | * |  | 0.7166574* |
| Female |  | 0.0693645* |  |  | -0.9551595 * |
| ICD-10 Code for criminal injustice involvement (z) |  | 0.0323654 | -0.2009082 * |  |  |
| ICD-10 Code for transportation barriers |  |  | * |  |  |
| ICD-10 Code for victim of crime, assault, or self-harm |  | * | 0.3408853* |  | 0.4276717 * |
| ICD-10 Code for food insecurity |  |  |  | 0.0457764* |  |
| ICD-10 Code for financial strain |  |  |  |  | 0.0094912 |
| ICD-10 Code for homelessness or housing instability | 0.3323964* |  |  | 0.1152699* | 0.0298672 |
| ICD-10 Code for unemployment |  |  |  |  |  |
| Language other than English preferred |  | 0.4338434* | * |  | * |
| More than 5 ED visits | 0.3955692* | 0.0517795* | 0.3752199* | 0.0967112* | 0.2416847 * |
| Neighborhood has high likelihood of social needs |  | * |  |  | 0.1971497 * |
| No emergency contact listed |  | * | * |  | * |
| No primary care visits | 0.0972018* |  | 0.211871 * |  | 0.013369 |
| No social security number on file |  |  |  |  | * |
| Notes mention financial instability |  |  | 0.0321961 * |  |  |
| Notes mention food insecurity |  | 0.1134791 |  | 0.075715 |  |
| Notes mention housing instability | 0.4009391* | 0.2208896* | 0.7491529 * | 0.5758405* |  |
| Notes mention legal involvement | 0.1188973* | 0.0309263* | 0.0170153 | 0.181797* | 1.2029482 * |
| Notes mention transportation barriers |  | 0.2509888* | 0.8392633 * | * |  |
| Patient Portal activated |  | * | * |  | * |
| Prior screener indicates financial insecurity |  | * |  | * |  |
| Prior screener indicates going without food |  | * |  |  |  |
| Prior screener indicates housing instability |  |  | * |  |  |
| Prior screener indicates transportation barriers |  |  | * |  |  |
| Previous scheduled encounter with social work |  |  | * |  |  |
| Previous scheduled visit with medical legal partnership |  |  | * |  |  |
| Public insurance (Medicare excluded) |  | 0.054857* | 0.1828912 * |  | 0.2290215 * |
| 75th percentile of total prescribed medications |  | * |  |  |  |
| Discharge to criminal justice settings | 0.0370782 |  | 0.2570737 |  |  |
| Total prior inpatient admissions (count) | 0.0001516 |  |  |  |  |
| Moved more than 3 times in past 12 months |  | 0.4663688 |  |  |  |

*Was included in the primary risk score modeling approach

Table A2. Performance of predictive models (using health information exchange data only) in classifying of adult emergency department patients with screening positive for the health-related social needs (HRSN) of housing instability, food insecurity, transportation barriers, and financial strain, Indianapolis, IN.

|  | AUC^1^ | Sensitivity | Specificity | + Likelihood ratio | Positive predictive value |
| --- | --- | --- | --- | --- | --- |
| Housing instability | 0.5838 |  |  |  |  |
| Medium^2^ |  | 30.0 | 85.9 | 2.128 | 54.3 |
| High |  | 18.8 | 94.0 | 3.103 | 72.5 |
|  |  |  |  |  |  |
| Food insecurity | 0.5945 |  |  |  |  |
| Medium^2^ |  | 56.6 | 58.7 | 1.370 | 65.7 |
| High |  | 16.0 | 94.6 | 2.982 | 83.5 |
|  |  |  |  |  |  |
| Transportation barriers | 0.6559 |  |  |  |  |
| Medium^2^ |  | 49.8 | 78.5 | 2.319 | 44.6 |
| High |  | 31.7 | 91.2 | 4.025 | 71.0 |
|  |  |  |  |  |  |
| Financial strain | 0.5856 |  |  |  |  |
| Medium^2^ |  | 27.6 | 89.1 | 2.539 | 36.5 |
| High |  | 5.7 | 99.1 | 6.222 | 61.9 |
|  |  |  |  |  |  |
| History of legal involvement | 0.6355 |  |  |  |  |
| Medium^2^ |  | 33.1 | 93.3 | 4.913 | 41.7 |
| High |  | 20.5 | 98.1 | 11.05 | 75.4 |

^1^ Area under the curve (for the overall risk score)

^2^ Medium category or greater score
