# Supplementary material for: Effectiveness of a clinical decision support system with prediction modeling to identify patients with health-related social needs in the emergency department: Study protocol
Source: PLoS One. 2025 May 12;20(5):e0323094. doi: 10.1371/journal.pone.0323094 (PMC12068607; doi:10.1371/journal.pone.0323094)
Supplement: S3 Appendix — (DOCX) [file pone.0323094.s005.docx]

**Appendix S3. Agile Implementation Vignettes**

**Food insecurity & screening focus**

**Patient / case description**: Mary is a single, 34 years old, female. She notes intermittent abdominal pain, which she describes as a dull ache. She usually gets her care from the Aspire Indiana community health center but admits to also having gone to Eskenazi and Community East emergency departments for similar conditions in the past couple of months.

**Use demonstration / action**: HealthDart has info on abdominal pain and information for those outside of IU health’s network.

**Resolution**: Contact the social work team and note the potential for food insecurity.

Admitting and patient

**Transportation discharge focus**

**Patient / case description**: One day John arrives at the emergency department with symptoms consistent with uncontrolled diabetes and hypertension. John is a 64-year old male from downtown Indianapolis. John took an Uber to get to the ED.

**Use demonstration / action**: Health Dart Demonstration -- At high risk for transportation barriers

**Resolution**: Referrals to social workers and care management can arrange transportation home and support John getting to his primary care visits after discharge.
