# Supplementary material for: Effectiveness of a clinical decision support system with prediction modeling to identify patients with health-related social needs in the emergency department: Study protocol
Source: PLoS One. 2025 May 12;20(5):e0323094. doi: 10.1371/journal.pone.0323094 (PMC12068607; doi:10.1371/journal.pone.0323094)
Supplement: S4 Appendix — (DOCX) [file pone.0323094.s006.docx]

**Appendix S4. Health-related social needs services**

Primary outcome #2: Percent of ED encounters that were referred for HRSN services. The numerator will be ED encounters with a referral to social worker, case management, community health workers, or related services within 24 hours of the ED encounter (see Appendix B). The denominator will be all eligible ED encounters (see **Inclusion criteria**, above).

Included services or data elements within 30 days of ED encounter:

- Any referral or order for social work, case management, nutrition counseling, financial planning, medical legal partnership assistance, patient navigation, and pharmacist consultation, or community health worker.
- Any appointment to the above services (scheduled, kept, no-show, or cancelled).
- Any referrals to “findhelp” services.
- NLP mention in note for social services.
