# Supplementary material for: Effectiveness of a clinical decision support system with prediction modeling to identify patients with health-related social needs in the emergency department: Study protocol
Source: PLoS One. 2025 May 12;20(5):e0323094. doi: 10.1371/journal.pone.0323094 (PMC12068607; doi:10.1371/journal.pone.0323094)
Supplement: S5 Appendix — (DOCX) [file pone.0323094.s007.docx]

**Appendix S4. Alternative models in case of data connectivity issues**

Our proposed prediction models used data from both EHR and HIE systems. If connections to the EHR data source were not possible due to technical or administrative reasons, we developed an alternative set of inputs and weights for the prediction risk score. These used data elements only available from the Indiana Network for Patient Care (HIE database). This included both structured data elements as well as factors extracted by natural language processing. The prediction target remained the same (i.e., binary indicators for positive results on each HRSN screening questionnaire) and the method for developing the risk score (i.e., Lasso models to identify a parsimonious set of predictors for logistic models, summing the unexponentiated post-selection model coefficients, and dividing the score into low, medium, and high categories). Additionally, we adjusted the thresholds so that the sensitivities (i.e., the percentage of true positives identified by the risk score) were more equivalent within each risk score level and overall.

Table A1. Alternative version of predictive models in classifying of adult emergency department patients with screening positive for the health-related social needs (HRSN) of housing instability, food insecurity, transportation barriers, financial strain and history of criminal justice involvement using only data available via health information exchange system, Indianapolis, IN.

| Features | Housing instability | Food insecurity | Transportation barriers | Financial strain | History of legal problems |
| --- | --- | --- | --- | --- | --- |
|  | $\beta$ | $\beta$ | $\beta$ | $\beta$ | $\beta$ |
| Address indicates homelessness | 0.2754314* | 0.3661188* | 0.6680268 * | 0.0638165* | 1.1474827* |
| Address matches known shelter address | 0.1110138* |  | 0.5825544 * | 0.0972929* |  |
| Address matches criminal justice location |  |  |  |  | 1.501319 |
| Age | -0.0007814 |  |  |  | 0.0047298* |
| Arrived via taxi at prior ED/hospitalization |  |  | * |  |  |
| Count of behavioral health encounters |  |  | * |  |  |
| Criminal justice associated payer | 0.4349765* |  | * |  | 0.7166574* |
| Female |  | 0.0693645* |  |  | -0.9551595 * |
| ICD-10 Code for criminal injustice involvement (z) |  | 0.0323654 | -0.2009082 * |  |  |
| ICD-10 Code for transportation barriers |  |  | * |  |  |
| ICD-10 Code for victim of crime, assault, or self-harm |  | * | 0.3408853* |  | 0.4276717 * |
| ICD-10 Code for food insecurity |  |  |  | 0.0457764* |  |
| ICD-10 Code for financial strain |  |  |  |  | 0.0094912 |
| ICD-10 Code for homelessness or housing instability | 0.3323964* |  |  | 0.1152699* | 0.0298672 |
| ICD-10 Code for unemployment |  |  |  |  |  |
| Language other than English preferred |  | 0.4338434* | * |  | * |
| More than 5 ED visits | 0.3955692* | 0.0517795* | 0.3752199* | 0.0967112* | 0.2416847 * |
| Neighborhood has high likelihood of social needs |  | * |  |  | 0.1971497 * |
| No emergency contact listed |  | * | * |  | * |
| No primary care visits | 0.0972018* |  | 0.211871 * |  | 0.013369 |
| No social security number on file |  |  |  |  | * |
| Notes mention financial instability |  |  | 0.0321961 * |  |  |
| Notes mention food insecurity |  | 0.1134791 |  | 0.075715 |  |
| Notes mention housing instability | 0.4009391* | 0.2208896* | 0.7491529 * | 0.5758405* |  |
| Notes mention legal involvement | 0.1188973* | 0.0309263* | 0.0170153 | 0.181797* | 1.2029482 * |
| Notes mention transportation barriers |  | 0.2509888* | 0.8392633 * | * |  |
| Patient Portal activated |  | * | * |  | * |
| Prior screener indicates financial insecurity |  | * |  | * |  |
| Prior screener indicates going without food |  | * |  |  |  |
| Prior screener indicates housing instability |  |  | * |  |  |
| Prior screener indicates transportation barriers |  |  | * |  |  |
| Previous scheduled encounter with social work |  |  | * |  |  |
| Previous scheduled visit with medical legal partnership |  |  | * |  |  |
| Public insurance (Medicare excluded) |  | 0.054857* | 0.1828912 * |  | 0.2290215 * |
| 75th percentile of total prescribed medications |  | * |  |  |  |
| Discharge to criminal justice settings | 0.0370782 |  | 0.2570737 |  |  |
| Total prior inpatient admissions (count) | 0.0001516 |  |  |  |  |
| Moved more than 3 times in past 12 months |  | 0.4663688 |  |  |  |

*Was included in the primary risk score modeling approach

Table 2. Performance of alternative version of predictive models (using health information exchange data only) in classifying of adult emergency department patients with screening positive for the health-related social needs (HRSN) of housing instability, food insecurity, transportation barriers, and financial strain, Indianapolis, IN.

|  | AUC^1^ | Sensitivity | Specificity | + Likelihood ratio | Positive predictive value |
| --- | --- | --- | --- | --- | --- |
| Housing instability | 0.5838 |  |  |  |  |
| Medium^2^ |  | 30.0 | 85.9 | 2.128 | 54.3 |
| High |  | 18.8 | 94.0 | 3.103 | 72.5 |
|  |  |  |  |  |  |
| Food insecurity | 0.5945 |  |  |  |  |
| Medium^2^ |  | 56.6 | 58.7 | 1.370 | 65.7 |
| High |  | 16.0 | 94.6 | 2.982 | 83.5 |
|  |  |  |  |  |  |
| Transportation barriers | 0.6559 |  |  |  |  |
| Medium^2^ |  | 49.8 | 78.5 | 2.319 | 44.6 |
| High |  | 31.7 | 91.2 | 4.025 | 71.0 |
|  |  |  |  |  |  |
| Financial strain | 0.5856 |  |  |  |  |
| Medium^2^ |  | 27.6 | 89.1 | 2.539 | 36.5 |
| High |  | 5.7 | 99.1 | 6.222 | 61.9 |
|  |  |  |  |  |  |
| History of legal involvement | 0.6355 |  |  |  |  |
| Medium^2^ |  | 33.1 | 93.3 | 4.913 | 41.7 |
| High |  | 20.5 | 98.1 | 11.05 | 75.4 |

^1^ Area under the curve (for the overall risk score)

^2^ Medium category or greater score
